# Hypercalciuria in Sarcoidosis: A Specific Biomarker With Clinical Utility

**DOI:** 10.3389/fmed.2020.568020

**Published:** 2020-10-29

**Authors:** Paolo Cameli, Carla Caffarelli, Rosa Metella Refini, Laura Bergantini, Miriana d'Alessandro, Martina Armati, Maria Dea Tomai Pitinca, Piersante Sestini, Stefano Gonnelli, Elena Bargagli

**Affiliations:** ^1^Respiratory Diseases Unit, Department of Medical, Surgical and Neurosciences Sciences, Siena University Hospital, Siena, Italy; ^2^Internal Medicine Unit, Department of Medical, Surgical and Neurosciences Sciences, Siena University Hospital, Siena, Italy

**Keywords:** sarcoidosis, calcium metabolism, biomarker, interstitial lung disease, specificity

## Abstract

**Background:** Changes in calcium metabolism are quite common in sarcoidosis: hypercalciuria is linked to a persistent clinical phenotype and more active disease. No data is yet available on the specificity of parameters of calcium metabolism as biomarkers for distinguishing different chronic interstitial lung diseases (ILD). Here we assessed calcium metabolism in an Italian population of sarcoidosis patients, which included a group with stage IV fibrotic disease, and compared the results with those of idiopathic pulmonary fibrosis (IPF) and chronic hypersensitivity pneumonitis (cHP) patients.

**Population and Methods:** We recruited sarcoidosis, IPF and cHP patients retrospectively. All patients were diagnosed through multidisciplinary discussion and were monitored at the Regional ILD Referral Centre in Siena. Clinical, radiological, functional, immunological and laboratory parameters were collected and entered in an electronic database for data analysis.

**Results:** A total of 305 patients (237 sarcoidosis, 40 IPF and 28 cHP) were enrolled. Sarcoidosis patients included a predominance of females and were significantly younger than IPF and cHP patients (*p* < 0.0001 for both). In the sarcoidosis population, 17 patients (7.2%) showed radiological evidence of lung fibrosis, according the Scadding classification; fibrotic disease was also confirmed by CT scan. Concerning calcium metabolism, sarcoidosis patients showed significantly higher serum and urinary concentrations of calcium than IPF and cHP patients (*p* = 0.0004 and *p* < 0.0001, respectively). These findings were also confirmed when comparing groups with fibrotic sarcoidosis, IPF and cHP (*p* = 0.0237 and *p* = 0.0138). According to receiver operating characteristics (ROC) curve analysis, urinary calcium showed better diagnostic accuracy than serum calcium in discriminating sarcoid and non-sarcoid lung fibrosis (AUC 0.7658 vs. 0.6205; *p* = 0.0026 vs. *p* = 0.1820).

**Discussion:** Our results confirmed that changes in calcium metabolism, particularly hypercalciuria, occur in a substantial percentage of patients with sarcoidosis. Higher serum and urinary concentrations of calcium were found than in IPF and cHP; the same results were observed when the comparison was limited to patients with fibrotic sarcoidosis, supporting the hypothesis that dysregulation of calcium metabolism may be a special feature of sarcoid granulomas. Hypercalciuria distinguished fibrotic sarcoidosis from IPF and cHP, suggesting that assessment of calcium metabolism may be useful in the diagnostic pathway of ILDs.

## Introduction

Sarcoidosis is a systemic disease included in the wide group of interstitial lung diseases (ILDs) that commonly require a multidisciplinary approach for diagnosis and clinical management. Due to its systemic nature, sarcoidosis needs to be assessed by a holistic approach that should include all possible localizations and expressions of disease. Changes in calcium metabolism, including hypercalcemia and hypercalciuria, are quite common (1–5). Their specific assessment is recommended and was recently endorsed by American Thoracic Society guidelines (6). Since 1960's, altered calcitriol production, parathyroid hormone (PTH) activity and sensitivity to Vitamin D have been described in sarcoidosis (7). Increased calcitriol levels cause absorption of calcium in the intestine and resorption from bone. Stimulated by interferon-γ (IFN- γ), tumor necrosis factor- α (TNF-α), interleukin-1 (IL1) and−2 (IL2), macrophages from sarcoid granulomas can spontaneously release 1,25-dihydroxy vitamin D, further boosting calcium resorption from the gastrointestinal tract and bone, leading to hypercalcemia and hypercalciuria (8, 9). Since abnormal resorption is associated with bone fragility and changes in bone mineral density, calcium metabolism is an issue in sarcoidosis, especially in patients requiring prolonged steroid treatment.

Changes in calcium metabolism and subsequent renal involvement (e.g., nephrocalcinosis) have been reported to have a prevalence of 5–60% in sarcoidosis patients (6, 10, 11). It has been clearly demonstrated that hypercalciuria and hypercalcemia are reliable negative prognostic factors, being associated with a chronic-persistent disease phenotype, high angiotensin-converting enzyme (ACE) levels, old age, hypergammaglobulinemia, and extrapulmonary sarcoid localizations (particularly in the spleen, bone and kidneys) (12–14). Our research group recently demonstrated a correlation between hypercalciuria and chitotriosidase concentrations, radiological evidence of severe lung involvement, deterioration of lung function (particularly concerning lung alveolar diffusion) and hepatosplenic disease (15), confirming the potential of this non-invasive and cost-sparing biomarker in routine clinical practice.

Little data is available on bone metabolism in other diffuse ILDs, despite the fact that osteoporosis is a common comorbidity in these patients (estimated prevalence >10%) (16). Moreover, since steroid therapy is the first-line therapy in many “inflammatory” ILDs, such as chronic hypersensitivity pneumonitis (cHP), assessment of bone and calcium metabolism may be useful in the management of these patients. Interestingly, in a cross-sectional study, our research group found a increased bone fragility with higher risk of vertebral fractures, irrespective of steroid therapy, in patients with idiopathic pulmonary fibrosis (IPF), suggesting that fibrotic lung disease *per se* may influence bone status and fracture risk (17).

Sarcoidosis is generally, but unwisely, viewed as a benign disease, although 9% of patients die from respiratory failure, particularly those with stage IV disease. Fibrotic lung sarcoidosis is observed in 5–15% of patients at presentation and is associated with poorer survival (18, 19). Isolated pulmonary stage IV sarcoidosis is a diagnostic challenge: differential diagnosis with respect to cHP and pneumoconiosis can be difficult due to similar clinical, immunological and radiological features in the end stage (20).

In the present study, we focused on changes in calcium metabolism in patients with granulomatous and non-granulomatous ILD, in order to evaluate their specificity as biomarkers in sarcoidosis and the clinical utility of differentiating stage IV sarcoidosis from other ILDs.

## Materials and Methods

### Study Population and Design

We recruited sarcoidosis, IPF and cHP patients retrospectively from the population monitored at the Siena Regional ILD Referral Center. Diagnosis was made according to international guidelines (6, 21); all patients underwent chest high resolution computed tomography (HRCT) for diagnostic purposes. All diagnoses were confirmed by multidisciplinary discussion: histological confirmation was available for 147 patients with sarcoidosis, six with IPF and four with cHP. For those patients in which histological sampling was not available or obtainable, diagnosis of sarcoidosis was made according to a multidisciplinary evaluation of clinical and radiological features, in order to exclude potential alternative diseases, as recently endorsed by American Thoracic Society guidelines (6). Sarcoid patients were also classified according to disease localization, as suggested by Genotype-Phenotype Relationship in Sarcoidosis project (GenPhenResA) (22). All patients underwent regular clinical evaluations at the Siena Referral Center for Osteoporosis. To be included in the study, patients had to provide serum and 24-h urine samples for assessment of calcium metabolism. Patients were specifically trained in 24-h urine collection. All patients were carefully evaluated in order to exclude potential comorbidities that may significantly influence serum and urinary biomarkers of calcium metabolism.

Patients were excluded if they were taking calcium or vitamin D supplements or drugs for osteoporosis. Demographic, radiological, immunological and functional data was collected from the medical records and entered in an electronic database.

Serum samples were also obtained from the sarcoidosis cohort to measure the disease-specific biomarkers chitotriosidase, and ACE.

All patients gave their informed consent to the study that was approved by the local Ethic committee. The study was conducted according to Declaration of Helsinki principles.

### Lung Function Tests

The following lung function measurements were recorded according to American Thoracic Society/European Respiratory Society (ATS/ERS) standards (23, 24), using a Jaeger body plethysmograph with corrections for temperature and barometric pressure: forced expiratory volume in 1 s (FEV1), forced vital capacity (FVC), FEV1/FVC, total lung capacity (TLC), residual volume (RV), lung diffusion capacity for carbon monoxide (DLCO) and DLCO/VA (alveolar volume).

### Chitotriosidase Assay

Human chitotriosidase activity was determined by a fluorimetric method using 22 μM 4-methylumbelliferyl β D-NNN-triacetylchitotriosidase (Sigma Chemical Co.) in citrate-phosphate buffer, pH 5.2; 100 μl substrate was incubated for 1 h at 37°C and the reaction was stopped with 1.4 ml 0.1 M glycine-NaOH buffer, pH 10.8. Fluorescence was read at 450 nm with a Perkin Elmer Victor X4 fluorimeter (excitation wavelength 365 nm). Serum chitotriosidase concentrations were expressed in mg/ml (normal values 1-44 mg/ml).

### Angiotensin Converting Enzyme Assay

ACE activity was measured previously described by a colorimetric method (FAR kit, FAR srl, Verona, Italy), widely used to determine ACE activity in serum, urine and tissues, as (16). The normal range of ACE concentrations is 30–80 U/l.

### Assessment of Calcium Metabolism

Serum concentrations of calcium (corrected for albumin), phosphate, total alkaline phosphatase and creatinine were measured using standard automated laboratory techniques. Urinary calcium, phosphate and creatinine were determined by a colorimetric method (Cobas C311 analyser, Roche Diagnostics, USA) in 24-h urine samples. Serum PTH was assessed by immunoradiometric assay (DiaSorin, Saluggia, Italy).

### Statistical Analysis

Data was expressed as mean ± standard deviations. Study variables were tested for normal distribution. The Mann-Whitney test or *t*-test were used for group comparisons on the basis of normality of data. The Kruskall-Wallis test was used to compare more than two groups. The Spearman test was used to find correlations. The analysis was run in GraphPad version 5.0.

## Results

### Clinical, Functional and Radiological Features

A total of 304 patients (236 sarcoidosis, 40 IPF and 28 cHP) were enrolled retrospectively in the study. Demographic, clinical, radiological and immunological data and functional parameters are reported in Table 1. As expected, sarcoidosis patients were younger, prevalently female and non-smokers, compared with IPF and cHP patients (*p* < 0.0001 for all comparisons). Concerning respiratory function, sarcoidosis patients showed normal lung volumes and diffusion capacity, while we observed mild restrictive impairment associated with moderate reduction in DLCO in IPF and cHP patients. Regarding radiological assessment, 17 sarcoidosis patients (7.2%) showed fibrotic lung disease, confirmed to be stage IV sarcoidosis by chest X-ray and HRCT. Despite their inclusion in stage 0 of disease according to Scadding classification, 91/99 sarcoidosis patients reported typical features of sarcoid lung involvement at HRCT; thus, only 8 patients showed (3.3%) an extrapulmonary disease phenotype, according to GenPhenResA assessment.

**Table 1:** Demographic features, functional parameters, radiological classification, and serum biomarkers' assessment of study population.

	**Sarcoidosis**	**IPF**	**cHP**	***p*-value**
*N*°	236	40	28	
Male (%)	92 (38.9)	33 (82.5)	17 (60.7)	<0.0001
Age (yrs)	56.1 ± 12.1	68 ± 8.4	66.8 ± 8.7	<0.0001
**Smoking history**				
Former (%)	85 (36)	34 (85)	15 (53.5)	<0.0001
Never (%)	151 (63.9)	6 (15)	13 (46.4)	<0.0001
**PFTs**				
FVC l (%)	3.5 ± 1 (106.7 ± 17.8)	2.6 ± 0.9 (73.3 ± 27.2)	2.4 ± 0.9 (75.7 ± 17.2)	<0.0001 <0.0001
FEV1 l (%)	2.6 ± 0.9 (98.3 ± 18.4)	2 ± 0.7 (76.4 ± 23.5)	1.9 ± 0.8 (76.2 ± 20.9)	<0.0001 <0.0001
FEV1/FVC	75.6 ± 7.4	78.8 ± 7.2	80.4 ± 10.4	0.0003^*^
DLCO %	80.1 ± 15.1	46.3 ± 17.1	54.2 ± 18.2	<0.0001
**GenPhenResA phenotypes**				
Abdominal (%)	17 (7.2)			
OCCC (%)	10 (4.2)			
Musculoskeletalcutaneous (%)	31 (13.1)			
Isolated pulmonary (%)	170 (72)			
Extrapulmonary (%)	8 (3.3)			
**Radiological assessment**^**¶**^				
CXR stage 0 (%)	99 (41.9)			
CXR stage 1 (%)	19 (8)			
CXR stage 2 (%)	51 (21.6)			
CXR stage 3 (%)	50 (21.1)			
CXR stage 4 (%)	17 (7.2)			
**Sarcoidosis biomarkers**				
Chitotriosidase (nmol/ml/h) [1–45 nmol/ml/h]	174.1 ± 90.6			
ACE (U/l) [30–80 U/l]	56.6 ± 22.8			

*If not otherwise reported, p-values referred to comparison between sarcoidosis and all other subgroups*.

¶ according to Scadding classification;

* *statistically significant difference between sarcoidosis and cHP patients; IPF, idiopathic pulmonary fibrosis; cHP, chronic hypersensitivity pneumonitis; PFTs, pulmonary function tests; FVC, forced volume capacity; FEV1, forced expiratory volume in the 1st s; DLCO, lung diffusion capacity for carbon monoxide; GenPhenResA, Genotype-Phenotype Relationship in Sarcoidosis; OCCC, ocular-cardiac-cutaneous-central nervous system; CXR, chest x-rays; AC, angiotensin-converting enzyme. Normal ranges of laboratory values are reported in square brackets*.

### Assessment of Calcium Metabolism

**Figure 1:**
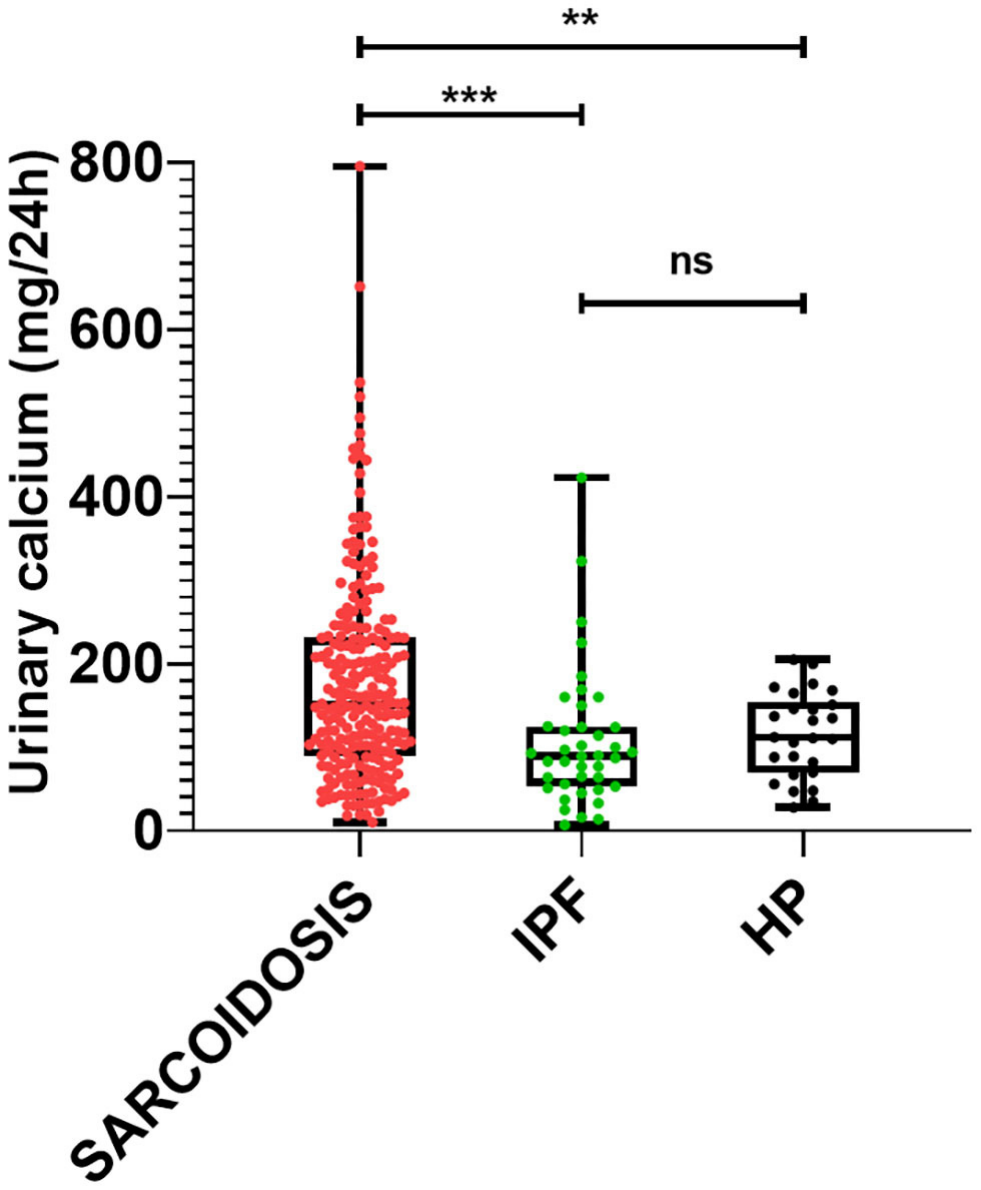
Comparison of 24-h urinary calcium concentrations among patients with sarcoidosis, IPF and cHP. Ns: not significant; ***p = 0.0007; **p = 0.003.

Table 2 shows the serum and urinary parameters of calcium metabolism measured in our center. We observed significantly higher levels of urinary calcium in sarcoidosis than in IPF and cHP patients (*p* = 0.0007) (Figure 1). This statistical difference remained significant when the comparison was limited to fibrotic stage IV sarcoidosis patients (*p* = 0.0138). Similarly, sarcoidosis patients showed significantly higher serum concentrations of calcium (whole population *p* = 0.0004; fibrotic group *p* = 0.0237).

**Table 2:** Calcium metabolism on serum and 24-h urinary sampling in sarcoidosis, IPF and cHP subgroups.

**Parameters**	**Sarcoidosis**	**IPF**	**cHP**	***p*-value**
**Serum**				
Calcium (mg/dl) [8.5–10.5 mg/dl)	9.58 ± 0.46	9.17 ± 0.51	9.29 ± 0.33	0.0004
Phosphate (mg/dl) [2.5–4.5 mg/dl]	3.42 ± 0.5	3.35 ± 0.42	3.57 ± 0.64	0.0957
Creatinine (mg/dl) [0.5–1.2 mg/dl]	0.94 ± 0.15	0.98 ± 0.13	0.98 ± 0.08	0.1265
Creatinine clearance (ml/min) [> 80 ml/min]	87.1 ± 40.3	88.5 ± 48.3	84.7 ± 35.9	0.8278
Alkaline phosphatase (U/l) [30–120 U/l]	66.1 ± 27.5	65 ± 19.3	58.8 ± 17.7	0.7145
**24 h-urine**				
Calcium (mg/24 h) [50–250 mg/24 h]	176.6 ± 120.1	111.4 ± 81.7	114 ± 53.4	0.0007
Phosphate (mg/24 h) [300–800 mg/24 h]	715.3 ± 307.4	758.7 ± 312.1	655.7 ± 312.1	0.1138
Creatinine (mg/24 h) [800–1,200 mg/24 h]	1,166.8 ± 506.7	1,219.1 ± 582.8	1,194.8 ± 501.4	0.7992

*IPF, idiopathic pulmonary fibrosis; cHP, chronic hypersensitivity pneumonitis. Normal ranges of values are reported in square brackets. P-value refers to sarcoidosis patients vs. all others*.

**Figure 2:**
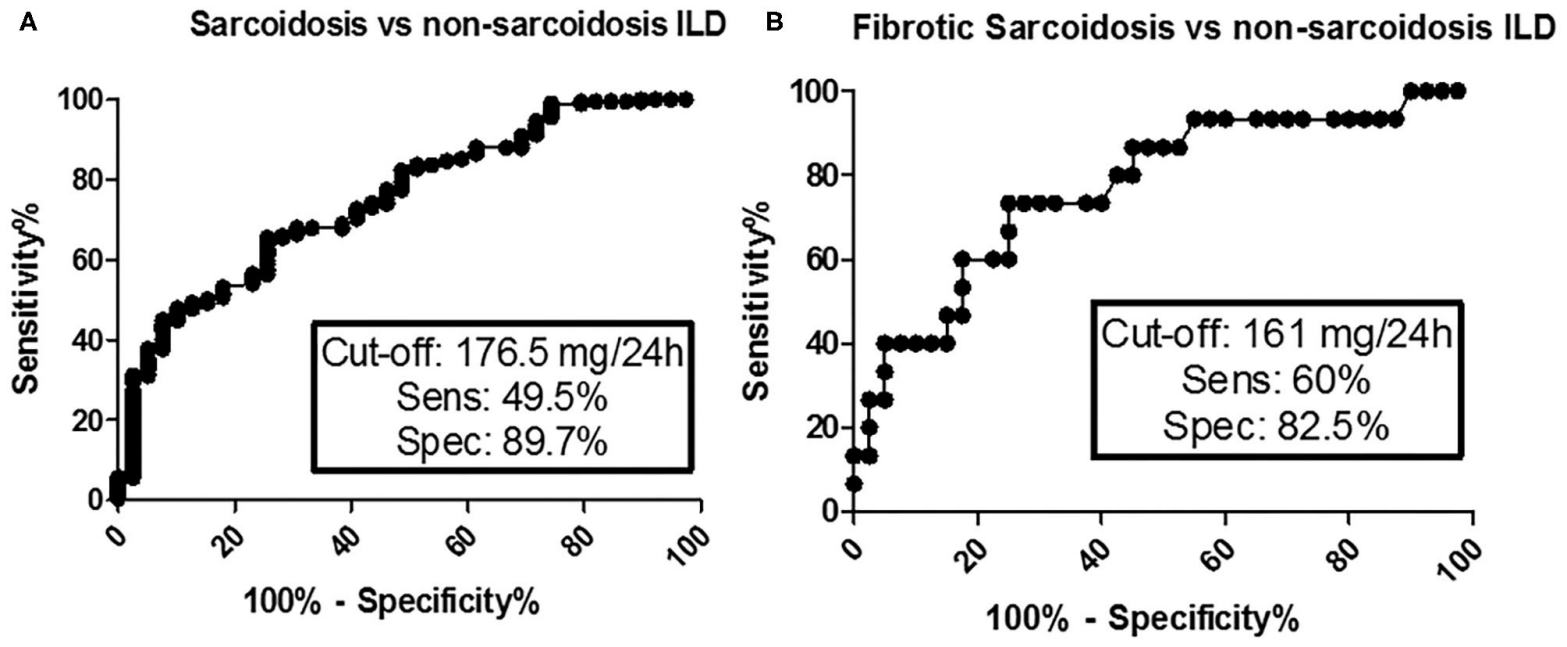
ROC analysis for diagnostic accuracy of urinary calcium between sarcoidosis and non-sarcoidosis ILD **(A)** and between fibrotic sarcoidosis and non-sarcoidosis ILD **(B)**.

ROC analysis was performed in order to evaluate the accuracy of serum and urinary calcium in discriminating sarcoidosis from IPF and cHP. Of the two biomarkers, urinary calcium showed better performance (AUC 0.7368, 95% CI 0.6573–0.8164, *p* < 0.0001 vs. AUC 0.6195, 95% CI 0.5090–0.7300, *p* = 0.01756), with a sensitivity of 49.5% and a specificity of 89.7% for a cut-off value of 176.5 mg/24 h (likelihood ratio 4.67). Comparing stage IV sarcoidosis patients with IPF and cHP patients, urinary calcium was confirmed to have a moderate-to-good accuracy, significantly better than serum calcium (AUC 0.7708, 95% CI 0.6284–0.9133, *p* = 0.0021 vs. AUC 0.6205, 95% CI 0.4558–0.7853, *p* = 0.1828) (Figure 2).

**Figure 3:**
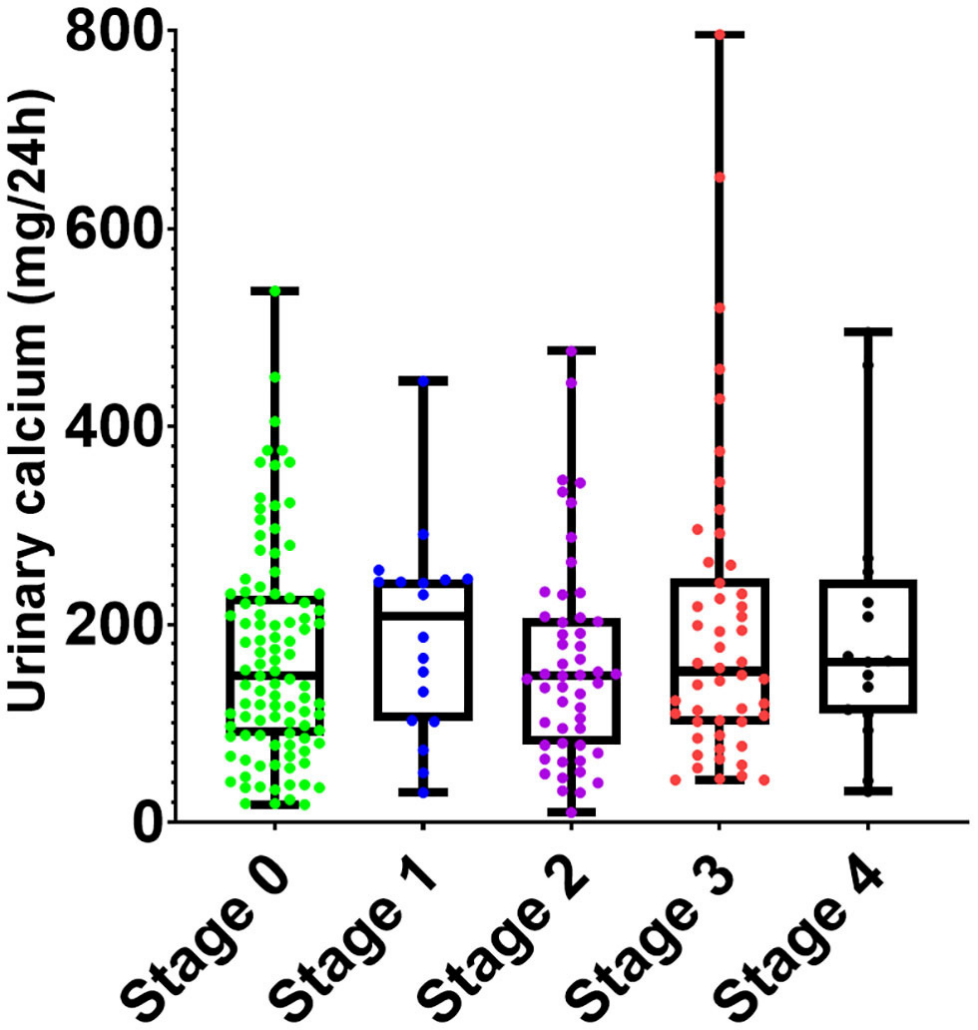
Comparison of 24-h urinary calcium concentrations among radiological stages of sarcoidosis. No statistically significant differences of urinary calcium concentration were observed among the subgroups.

No significant differences of serum and urinary parameters of calcium metabolism were found among different clinical phenotypes or radiological stages in the sarcoidosis group (Figure 3).

### Correlations

We observed a significant direct correlation between urinary calcium and chitotriosidase activity in patients with sarcoidosis (*r* = 0.2564, *p* = 0.009), but not with ACE (*r* = 0.0938, *p* = 0.1535). Urinary calcium was also inversely correlated with DLCO (*r* = −0.1428, *p* = 0.0373) in patients with sarcoidosis, but not in IPF and cHP patients (*p* = 0.3893, and *p* = 0.8091).

## Discussion

The aim of the present study was to evaluate the potential of parameters of calcium metabolism in the diagnostic algorithm of diffuse ILDs. Hypercalciuria and hypercalcemia are quite common anomalies in sarcoidosis patients and need to be addressed to prevent severe complications or chronic organ failure (e.g., ventricular arrhythmia, nephrolithiasis, nephrocalcinosis, chronic renal failure). The clinical features of calcium dysregulation in sarcoidosis have been repeatedly described (2, 15, 25), as well as its underlying pathophysiological processes: however, there are still many concerns about how it should be treated and monitored (26). The guidelines published recently by the ATS endorsed the utility of assessing calcium metabolism in sarcoidosis, recommending serum calcium testing at diagnosis and follow-up to screen for specific alterations (6).

Our results showed that urinary and serum calcium are both significantly higher in sarcoidosis than in IPF and cHP. Twenty-four hour urinary calcium (not serum calcium assay) showed promising accuracy in discriminating sarcoidosis from the other two ILDs. To our knowledge, the specificity of hypercalcemia or hypercalciuria in the setting of ILDs has never been researched, and no studies have ever investigated the potential of alterations in calcium metabolism as an indicator of non-sarcoid lung fibrosis. Sarcoid-related calcium alterations are possibly determined by overexpression of 1-alpha-hydroxylase and parathyroid hormone-related proteins by granulomatous macrophages (26–28), but it is not known whether this dysregulation is specific to sarcoidosis or also occurs in other granulomatous ILDs, such as cHP. Since no differences were observed between IPF and cHP patients, our results suggest that changes in calcium metabolism may be related to sarcoid granuloma activity. These assumptions are also supported by the significant correlation between urinary calcium and chitotriosidase, a macrophage-derived chitinase, specifically linked to sarcoidosis activity and severity. This correlation was previously reported by our research group and is probably determined by the aberrant activation of macrophages in sarcoid granulomas (12). Our results therefore confirm that urinary calcium is related to disease activity, although it appeared to be less sensitive than chitotriosidase, if compared with the studies available in literature (12, 29, 30). It is still unknown whether specific patterns of macrophage activation, linked to different cytokine overexpression patterns, may lead to different clinical features or disease phenotypes. However, another finding of the present study, in line with previous reports, was that urinary calcium is also inversely correlated with DLCO percentages (15). As DLCO was substantially normal in our sarcoidosis cohort, these findings suggest that urinary calcium could be useful as an early indicator of a chronic-progressive sarcoid phenotype, leading to lung fibrosis. These results are interesting and worthy of further research in large prospective cohorts.

The accuracy of urinary calcium for differential diagnosis was also confirmed when the comparison was limited to stage IV fibrotic sarcoidosis and IPF-cHP subgroups. Fibrotic sarcoidosis may have clinical onset and progression indistinguishable from IPF and cHP, with restrictive functional impairment and similar HRCT features in many patients. Stage IV sarcoidosis, like HP, is regarded as a “great mimicker” and may therefore be a diagnostic challenge in the differential diagnosis of ILDs (20, 31). Since no biomarkers have yet been approved to distinguish these ILDs (32), changes in calcium metabolism may be suggested as a bioindicator specific to sarcoidosis among ILDs. Our findings suggest that urinary calcium assessment may be useful to discriminate end-stage sarcoidosis from other fibrotic ILDs and as a biomarker in a multidisciplinary setting. It has the advantage of being simple, non-invasive and economical.

Our study has some limitations: first, the sample size, though relevant for rare diseases such as sarcoidosis, IPF and cHP, is not sufficient to properly assess the reliability of our results, as well as the monocentric nature of the study. Second, the retrospective design is intrinsically prone to referral and reporting bias, that may significantly influence the analysis and the interpretation of data. Third, due to the lack of data contemporary to urinary sampling, we didn't include in the analysis serum 25-OH and 1, 25-OH vitamin D concentrations. Considering the prominent role of vitamin D in calcium metabolism, future and prospective studies will address the concentration of 25-OH and 1-25-OH forms to further clarify their potential value on this field.

In conclusion, in this study we found a significant increase in serum and urinary concentrations of calcium in sarcoidosis patients with respect to IPF and cHP patients. The finding sustains the specificity of changes in calcium metabolism in this granulomatous lung disease. Urinary calcium revealed good specificity for fibrotic (stage IV) sarcoidosis, suggesting that it has potential as a biomarker for the differential diagnosis of ILDs and in the estimation of sarcoid disease activity.

## Data Availability Statement

The raw data supporting the conclusions of this article will be made available by the authors, without undue reservation.

## Ethics Statement

The studies involving human participants were reviewed and approved by Comitato Etico Area Vasta Sud Est (C.E.A.V.S.E.). The patients/participants provided their written informed consent to participate in this study.

## Author Contributions

PC, CC, RR, SG, and EB: conception, study design, interpretation of results, and writing of the manuscript. LB, MT, MA, and Md'A: data acquisition and analysis, revision of the study, and interpretation of results. PC, CC, and PS: statistical analysis and revision of the study. All authors approved the final version of the study and agreed to be accountable for all aspects of the work in ensuring that questions related to the accuracy or integrity of any part of the work are appropriately investigated and resolved.

## Conflict of Interest

The authors declare that the research was conducted in the absence of any commercial or financial relationships that could be construed as a potential conflict of interest.

## Abbreviations

DL_co_: diffusing capacity of the lung for CO
FVC: forced vital capacity
FEV1: forced expiratory volume in 1 second
IPF: idiopathic pulmonary fibrosis, cHP, chronic hypersensitivity pneumonitis
HRCT: high resolution computed tomography.
